# Anti-Inflammatory and Antioxidant Properties of Squalene in Copper Sulfate-Induced Inflammation in Zebrafish (*Danio rerio*)

**DOI:** 10.3390/ijms24108518

**Published:** 2023-05-10

**Authors:** Peng Zhang, Naicheng Liu, Mingyang Xue, Mengjie Zhang, Zidong Xiao, Chen Xu, Yuding Fan, Wei Liu, Junqiang Qiu, Qinghua Zhang, Yong Zhou

**Affiliations:** 1 Yangtze River Fisheries Research Institute, Chinese Academy of Fishery Sciences, Wuhan 430223, China; 2 Key Laboratory of Exploration and Utilization of Aquatic Genetic Resources, Ministry of Education, Shanghai Ocean University, Shanghai 201306, China; 3 National Pathogen Collection Center for Aquatic Animals, Shanghai Ocean University, Shanghai 201306, China

**Keywords:** squalene, zebrafish (*Danio rerio*), CuSO_4_, anti-inflammation, antioxidation

## Abstract

Long-term or excessive oxidative stress can cause serious damage to fish. Squalene can be added to feed as an antioxidant to improve the body constitution of fish. In this study, the antioxidant activity was detected by 2,2-diphenyl-1-acrylhydrazyl (DPPH) test and fluorescent probe (dichloro-dihydro-fluorescein diacetate). Transgenic *Tg* (*lyz*: *DsRed2*) zebrafish were used to evaluate the effect of squalene on CuSO_4_-induced inflammatory response. Quantitative real-time reverse transcription polymerase chain reaction was used to examine the expression of immune-related genes. The DPPH assay demonstrated that the highest free radical scavenging exerted by squalene was 32%. The fluorescence intensity of reactive oxygen species (ROS) decreased significantly after 0.7% or 1% squalene treatment, and squalene could exert an antioxidative effect in vivo. The number of migratory neutrophils in vivo was significantly reduced after treatment with different doses of squalene. Moreover, compared with CuSO_4_ treatment alone, treatment with 1% squalene upregulated the expression of *sod* by 2.5-foldand *gpx4b* by 1.3-fold to protect zebrafish larvae against CuSO_4_-induced oxidative damage. Moreover, treatment with 1% squalene significantly downregulated the expression of *tnfa* and *cox2*. This study showed that squalene has potential as an aquafeed additive to provide both anti-inflammatory and antioxidative properties.

## 1. Introduction

Reactive oxygen species (ROS) and free radicals are involved in several diseases, including metabolic, and infectious diseases [1,2]. ROS can affect apoptosis and the immune system, and enhance the inflammatory response. Therefore, ROS are believed to cause many diseases and are a major factor in aging [3]. An imbalance between ROS production and the counteracting antioxidant system allows ROS to interact with and cause damage to proteins, lipids, and DNA. This imbalance is referred to as oxidative stress [2]. Inflammation and oxidative stress are interdependent mechanisms [4]. Moreover, oxidative stress might provoke inflammatory responses that can further enhance oxidative stress [5]. 

Zebrafish is a widely used animal model and has a variety of transgenic or mutant fish lines that can study human and aquatic animal diseases [6]. At the same time, zebrafish has a variety of inflammatory models [6]. When fish are stressed, the production of oxygen free radicals in tissues or cells increases [7,8]. This leads to a large amount of ROS in tissues or cells, causing oxidative damage [9]. Long-term or excessive oxidative stress can cause damage to fish, such as slow growth and development, decreased immune function, and increased disease incidence [8]. Oxidative stress damage to fish can reduce the quality of aquatic products and the feed conversion rate, causing serious economic losses to the aquaculture industry [10]. Therefore, it is of great significance to find natural products with antioxidant function in the study of inflammation and oxidation in fish. The zebrafish immune system is very similar, at the molecular and cellular levels, to that in humans [11]. In addition, the adaptive immune system in zebrafish is morphologically and functionally mature only at 4 to 6 weeks post-fertilization, and only innate immunity exists in embryos [12]. Oxidative and inflammatory responses can be induced and visualized easily in the early stages of zebrafish development, for example by transection of the tail fin, copper sulfate (CuSO_4_), and lipopolysaccharide (LPS) immersion [13]. Excessive inorganic copper in the environment destroys the copper balance in zebrafish, raising serum copper levels, and triggering the oxidative stress response, leading to an oxidative stress damage-mediated inflammatory response [14]. At the same time, oxidative stress induces the death of hair cells in the lateral line neuromasts of zebrafish larvae, causing the migration of immune cells [14].

Squalene is an unsaturated hydrocarbon containing six isoprene double bonds [15], belonging to the terpenoids [16]. Squalene, originally isolated from sharks, is a natural product that is widely distributed in nature [17]. Subsequently, it was found that squalene occurred in high concentrations in shark liver, vegetable oils, and in the stomach oil of birds [17]. Squalene is synthesized in the human liver and skin and plays an important role in cholesterol synthesis [18]. Later studies found that squalene had potent cytoprotective and antitumor activities [19,20,21]. Over recent decades, squalene had been used in the field of human cosmetic and medicine because of its antioxidant and anticancer activities, because tested compound treatment relieved oxidative stress in epithelial cells and reduced oxidative damage in rat models [22,23]. Squalene can improve dextran sulfate sodium (DSS)-induced acute colitis in mice [24]. It can confer survival advantage to treated animals and reduce the content of Malonaldehyde (MDA) in models of endotoxemia [25]. In human mammary epithelial cells (MCF10A), squalene reduced intracellular ROS levels, prevented H_2_O_2_-induced oxidative damage, and prevented oxidative DNA damage [26]. The generation of ROS can be avoided by the chelating of metal ions. Previous studies have shown that olive oil (rich in squalene) with a concentration of less than 100 μg/mL has a low chelating ability to Fe^2+^ [27]. Squalene has also been found to augment the secretion of interleukin 1 beta (IL-1β) by macrophages, thus suggesting a role for sebaceous glands in modulating innate immune responses via their secreted lipids, thus contributing to the development of inflammatory acne [28]. The anticancer, antioxidant, drug carrier, and other characteristics of squalene have resulted in its wide use to control and treat diseases [23]. In addition, squalene’s role in the field of functional food applications is also expanding [29,30], but there have been few studies on the effect of squalene application in fish. Therefore, in the present study, zebrafish were used as an experimental model to study the effects of squalene on anti-inflammation and antioxidant, with the aim of providing a theoretical basis for the preparation of fish food additives.

## 2. Results 

### 2.1. Copper Chelating Activity (CCA) of Squalene

**Figure 1:**
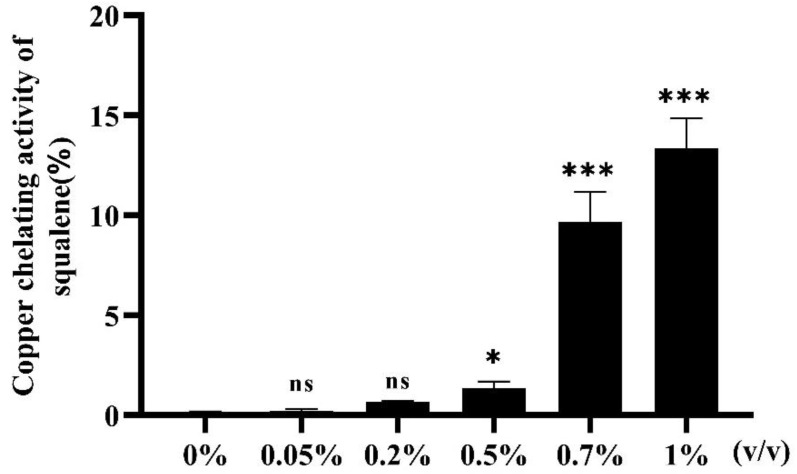
Copper chelating activity of squalene. Each bar represents the mean ± S.D. for three different experiments performed in triplicate, ns, not significant, * *p* < 0.05, *** *p* < 0.001 compared with the 0% group (control group).

The metal chelating ability of squalene toward Cu^2+^ was verified by using pyrocatechol violet (PV) as a chromogenic agent. At the range of concentrations used in the experiment, the metal chelating ability of squalene increased in a concentration-dependent manner (Figure 1). There was no significant difference between the 0.05% and 0.2% squalene groups and the 0% group. Compared with the 0% group, the 0.5%, 0.7%, and 1% squalene group results were significantly different. In addition, 1% squalene had the highest CCA, which was only 13.3%. This result confirmed that the CCA of squalene was weak.

### 2.2. 2,2-Diphenyl-1-Picrylhydrazyl (DPPH) Radical Scavenging Activity of Squalene

**Figure 2:**
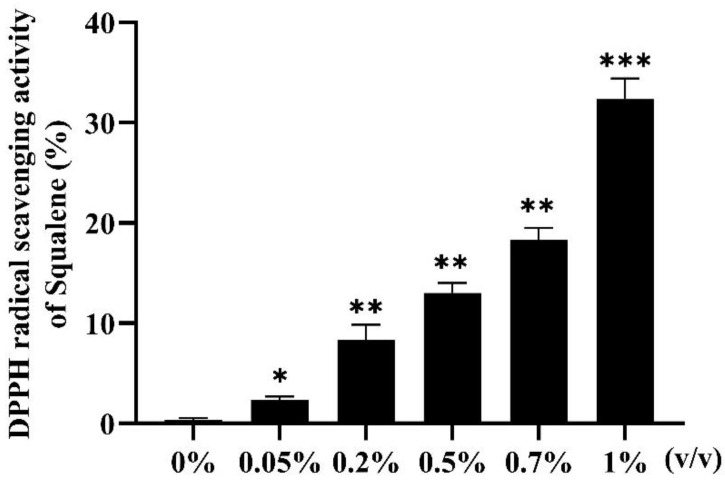
DPPH radical scavenging activity of squalene. Each bar represents the mean ± S.D. for three different experiments performed in triplicate, * *p* < 0.05, ** *p* < 0.01, *** *p* < 0.001 compared with the 0% group (control group). DPPH—2,2-Diphenyl-1-picrylhydrazyl.

The in vitro antioxidant activity of squalene was determined by measuring its DPPH radical scavenging activity. Compared with that of the 0% group, the results of each experimental group were significantly different (Figure 2). At the range of concentrations used in the experiment, the scavenging activity of squalene increased in a dose-dependent manner. DPPH radical scavenging activity of 1% squalene was the highest (32%). The results showed that squalene had certain DPPH free radical scavenging ability.

### 2.3. In Vivo Antioxidant Effect of Squalene

**Figure 3:**
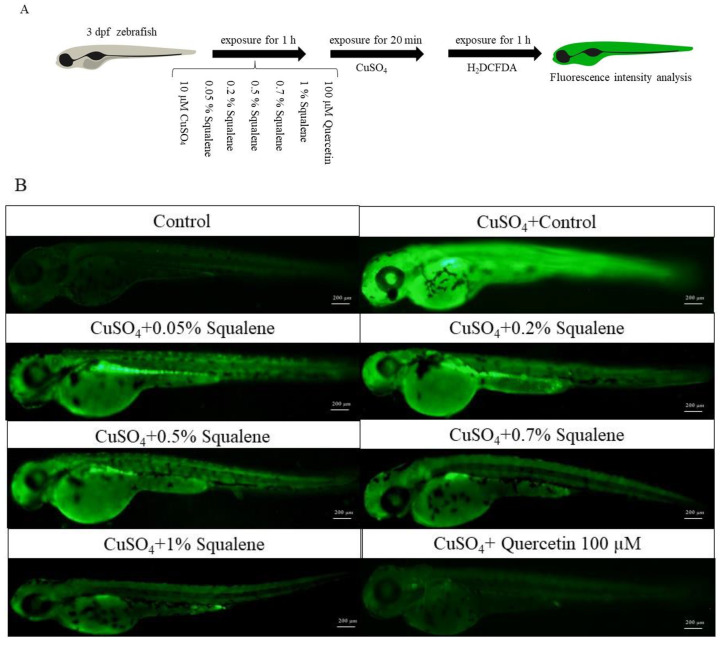

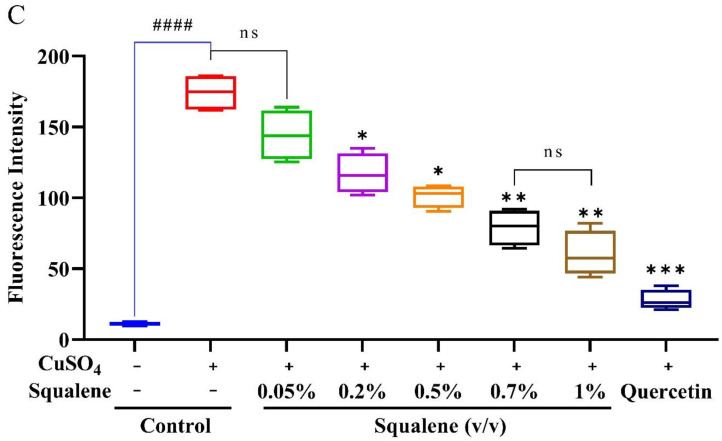
Squalene inhibits CuSO_4_-induced reactive oxygen species production in zebrafish larvae. (**A**) Flowchart of the experiments (**B**) Fluorescence images of representative detections of reactive oxygen species, scale bars represent 200 µm. (**C**) Fluorescence intensity of juvenile zebrafish compared with that of the group using CuSO_4_ alone, the data were presented as medians for four different larvae. #### *p* < 0.001, compared with the control group. ns, not significant. * *p* < 0.05, ** *p* < 0.01, *** *p* < 0.001, compared with the CuSO_4_ group. H_2_DCFDA—dichlorodihydrofluorescein diacetate.

The fluorescent probe dichloro-dihydro-fluorescein diacetate (H_2_DCFDA) was used as a ROS indicator to evaluate the effect of squalene on ROS production in zebrafish (Figure 3A). Compared with that in the control group, the fluorescence intensity (FI) of the CuSO_4_ treatment group increased (Figure 3B,C). This indicated that CuSO_4_ induced more ROS in zebrafish, and the experimental model was established. The FI of the 100 µM quercetin group was significantly decreased compared with that in the CuSO_4_ group (Figure 3B,C). Similar to quercetin, the mean FI of ROS was decreased in the 0.2%, 0.5%, 0.7%, and 1% squalene-treated groups (Figure 3B,C). No significant difference was found between the 0.7% and 1% squalene-treated groups. After treatment with squalene, the FI decreased with the increase in squalene concentration. However, the effect of 0.05% squalene treatment on the production of reactive oxygen was not significant. In summary, squalene inhibited ROS generation in a dose dependent manner. At the lowest concentration (0.05% squalene), the effect of treatment was not significant.

### 2.4. Effect of Squalene on the Expression of Antioxidant Genes sod and gpx4b

**Figure 4:**
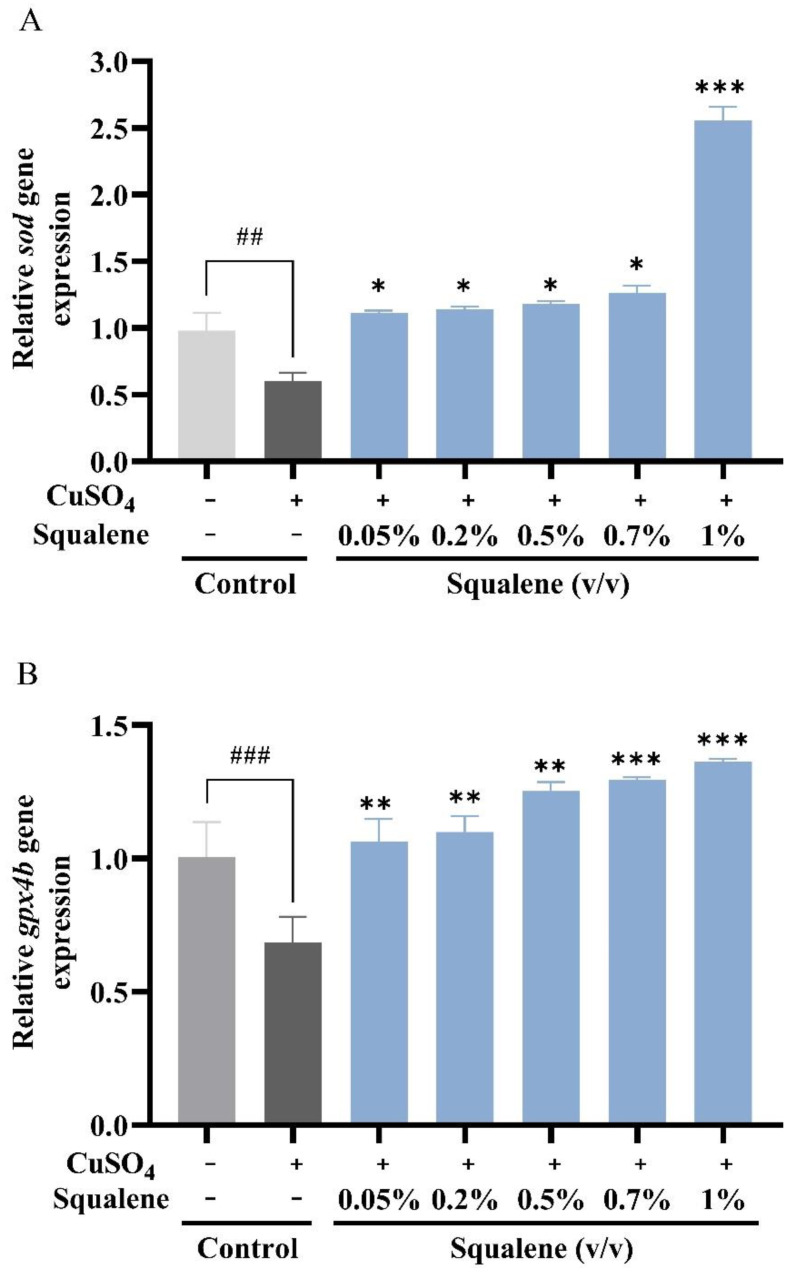
The relative expression of antioxidant-related genes relative to *β-actin* in zebrafish larvae treated with CuSO_4_ and squalene for 24 h. (**A**) The expression of *sod* gene relative to *β-actin* (**B**) The expression of *gpx4b* gene relative to *β-actin*, the data were expressed as the mean ± SD, three biological replicates, ## *p* < 0.05, ### *p* < 0.01, compared with the control group. * *p* < 0.05, ** *p* < 0.01, *** *p* < 0.001 compared with CuSO_4_ alone treatment group. *sod*—superoxide dismutase; *gprx4b*—glutathione peroxidase 4b.

The expression levels of *sod* (encoding superoxide dismutase) and *gpx4b* (encoding glutathione peroxidase 4b) were assessed using RT-qPCR. Compared with those in the control group, the expression levels of *sod* and *gpx4b* in the CuSO_4_ alone treatment group were significantly reduced (Figure 4A,B). After combined treatment with squalene for 24 h, the expression of *sod* in the 1% squalene treatment group was upregulated by 5-fold. The *sod* expression levels in the remaining experimental groups were increased by 2-fold (Figure 4A). The difference between all the experimental groups and the CuSO_4_ alone treatment group was statistically significant. The effect of squalene on the expression of *gpx4b* was dose-dependent (Figure 4B).

### 2.5. In Vivo Neutrophil Recruitment Assay

**Figure 5:**
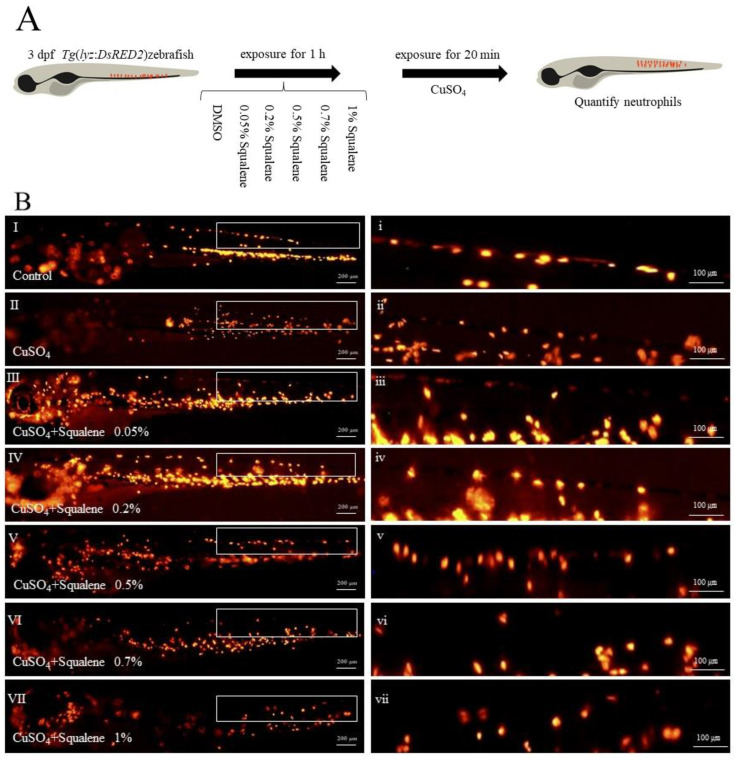

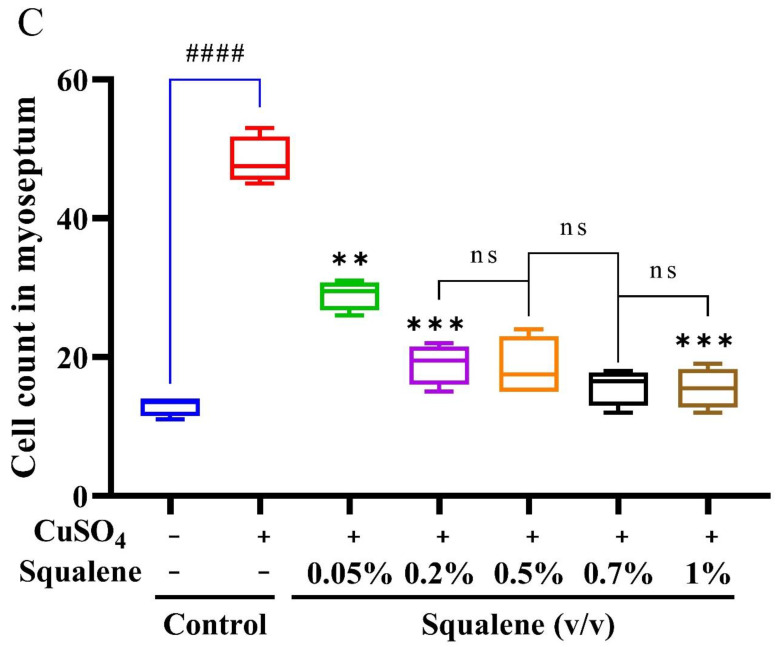
Effect of squalene on CuSO_4_-induced inflammatory response in zebrafish. (**A**) Flowchart of the experiments (**B**) Neutrophil migration in zebrafish after treatment, scale (**I**–**VII**) bars represent 200 µm, scale (**i**–**vii**) bars represent 100 µm. (**C**) Quantification of the number of neutrophils aggregated to the lateral line after CuSO_4_ treatment, the data are presented as medians for four different larvae. Data are expressed as the mean ± SD. #### *p* < 0.001, compared with the control group. ns, not significant. ** *p* < 0.01, *** *p* < 0.001, compared with the CuSO_4_ alone treatment group, *n* = 4. dpf—days post-fertilization; DMSO—dimethyl sulfoxide.

The CuSO_4_ inflammation model was constructed using transgenic zebrafish *Tg* (*lyz*: *DsRED2*) to verify the effect of squalene on neutrophil migration after the zebrafish were stimulated by Cu^2+^ (Figure 5A). In the control group, neutrophils were localized to the caudal hematopoietic tissue in the ventral trunk and tail (Figure 5B(I)). In contrast, in fish treated with CuSO_4_, neutrophils migrated to the horizontal midline and formed clusters near the lateral line neuromasts (Figure 5B(II)). However, pretreatment with squalene solution (0.05%, 0.2%, 0.5%, 0.7%, and 1%) significantly inhibited neutrophil migration to inflammatory sites (Figure 5C). The results for all squalene pretreatment groups were significantly different when compared with the CuSO_4_ alone treatment group (Figure 5C). There was no significant difference between 0.2%, 0.5%, 0.7%, and 1% groups.

### 2.6. Effect of Squalene on Expression of tnfa and cox-2

**Figure 6:**
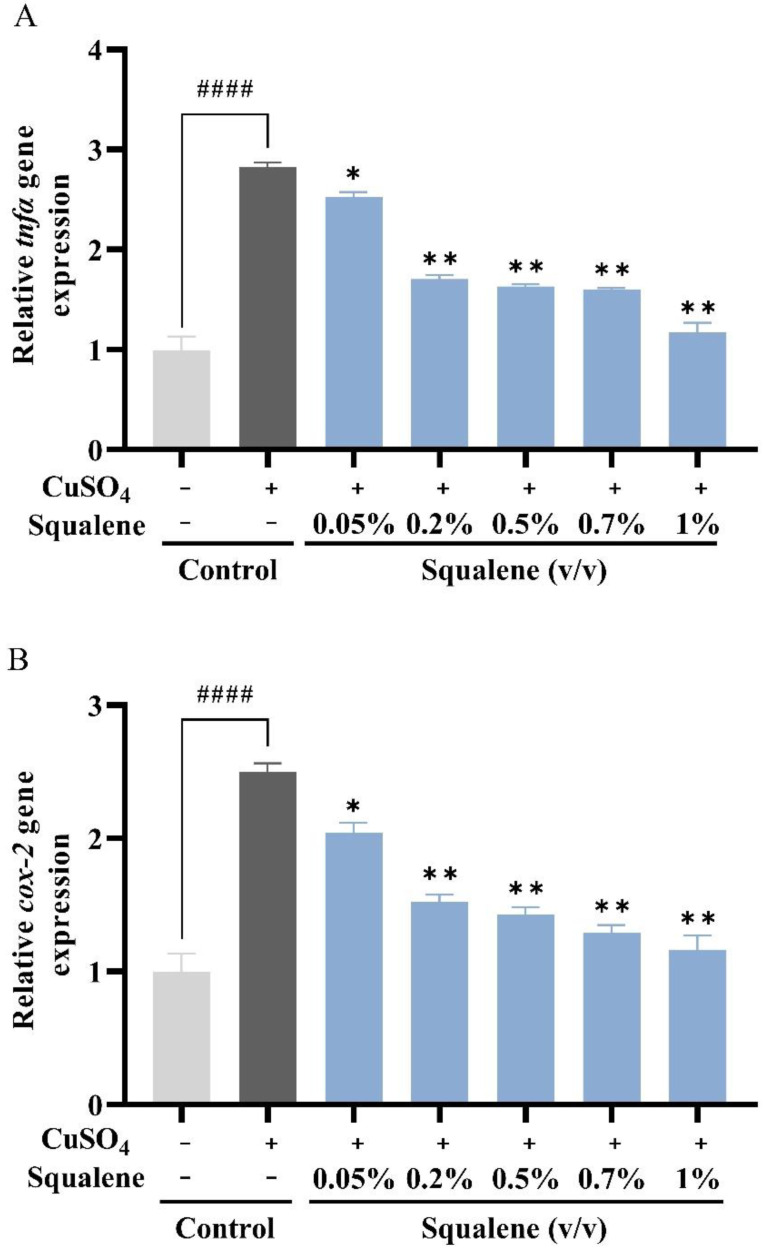
The expression of immune response-related genes relative to *β-actin* in zebrafish larvae after CuSO_4_ stimulation and squalene treatment. (**A**) The expression of *tnfa* gene relative to *β-actin* (**B**) The expression of *cox-2* gene relative to β-actin. The data were expressed as the mean ± SD, three biological replicates, #### *p* < 0.001, compared with the control group. * *p* < 0.05, ** *p* < 0.01 compared with CuSO_4_ alone treatment group. *tnfa*—tumor necrosis factor alpha; *cox-2*—cyclooxygenase 2.

The expression levels of *tnfa* (encoding tumor necrosis factor alpha) and *cox-2* (encoding cyclooxygenase 2) were significantly upregulated in zebrafish after CuSO_4_ only stimulation, by more than 2.5 times (Figure 6A,B). Compared with the CuSO_4_ alone treatment group, the expression levels of *tnfa* and *cox*-*2* were downregulated after treatment with squalene for 24 h, in a dose-dependent manner (Figure 6A,B). The most marked inhibition was achieved using 1% squalene.

## 3. Discussion

The CuSO_4_-induced inflammation model can be established by simply adding the compound CuSO_4_ into the zebrafish larvae culture medium. The accumulation of neutrophils in the neuromasts is one of the most frequently used indicators for the level of inflammation in CuSO_4_-induced inflammation model and has been applied to assess the effect of known anti-inflammatory drugs [6]. Neutrophils migrate to inflammatory foci and can be observed in zebrafish [31,32]. Therefore, in this study, CuSO_4_ was selected to stimulate oxidative stress and induce inflammation. The established zebrafish inflammation model was used to verify the anti-inflammatory and antioxidant effects of squalene on zebrafish. This study has shown that inflammation occurs after zebrafish are stimulated, including increased ROS content and neutrophil migration to the neuromast. In this study, the use of squalene reduced inflammation and enhanced the anti-inflammatory and antioxidant capacities of zebrafish.

### 3.1. Antioxidant Effect of Squalene on Zebrafish

To assess the antioxidant activity of squalene, we first used an in vitro DPPH assay. The DPPH radical scavenging assay is widely used to determine the antioxidant activity of natural compounds because of its simplicity, sensitivity, comparable nature, and reproducibility [33,34,35]. The extract of *Prunus mume* (squalene-rich) had a good DPPH free radical scavenging ability, and its ethanol extract had the highest antioxidant activity (96.08–97.71%), whereas its water extract had the lowest antioxidant activity (76.09%) [36]. In our study, the free radical scavenging rate of the 1% squalene treatment group was 32% (Figure 2). With the increase in squalene concentration, the scavenging rate of DPPH free radical increased. This indicated that squalene can scavenge DPPH free radicals and squalene has certain antioxidant activity.

Although the determination of the antioxidant capacity by the DPPH method in vitro is simple and convenient, it cannot replace animal experiments in vivo [37,38]. Therefore, this study used zebrafish to establish an inflammatory model to test the antioxidant and anti-inflammatory effects of squalene. CuSO_4_ stimulated the generation of ROS, and ROS production could induce an inflammatory reaction [39,40,41]. In addition, CuSO_4_ could interfere with the expression of antioxidant genes, such as *sod* and *gpx* [42,43]. Therefore, this study mainly studied the effects of squalene treatment on ROS production and antioxidant gene expression in vivo. To eliminate the factors affecting the experimental results due to the complexation of Cu^2+^ with squalene during incubation, first, we tested the CCA of squalene, which showed that the complexation rate of 1% squalene to Cu^2+^ was 13.3% (Figure 1). The CCA of squalene is very low and would not affect the subsequent experimental results. Squalene can reduce AAPH (2,2′-azobis-2-methyl-propanimidamide, dihydrochloride) and H_2_O_2_-induced oxidative stress in Vero cells and reduce UV-induced intracellular ROS levels [44]. Squalene-based PLGA NPs (ploy lactic-co-glycolic acid nanoparticles) effectively reduced ROS levels in normal mouse hepatocytes [45]. Then, an in vivo experimental model was established by soaking zebrafish in CuSO_4_ to produce oxidative stress, permitting the verification of the antioxidant effect of squalene on zebrafish. ROS generation was then evaluated using the dye H_2_DCFDA [46,47,48]. The results showed that squalene treatment could effectively reduce ROS produced in zebrafish in a dose-dependent manner (Figure 3A,B). The 0.7% and 1% squalene treatment groups showed the best effect, with no significant differences between these two groups (Figure 3B). This demonstrated that squalene had a significant antioxidant effect, which could reduce the production of ROS in zebrafish larvae and protect zebrafish from stress.

Antioxidant enzymes include glutathione peroxidase (GPX, GSH-Px) and superoxide dismutase (SOD) [49,50]. GSH-Px plays a crucial role against oxidative stress, turning toxic substances into innocuous products by scavenging their free radicals [51,52]. GPx converts H_2_O_2_ and lipid peroxides into water and lipid alcohols to exert similar antioxidant effects [53]. Therefore, SOD and GSH-Px can reflect the body’s ability to resist oxidative damage [54]. In the oxidative damage model of zebrafish embryos induced by cadmium (Cd), a tree peony seed protein hydrolysate could inhibit Cd-induced oxidation by upregulating the expression of *sod* and *gpx* mRNA [55]. Rats fed with 2% squalene showed a significant decrease in lipid peroxidation and a significant increase in SOD and CAT activity, indicating the antioxidant properties of squalene in experimental induction [56]. Administration of squalene in rats improved GPx activity and the GSH level in heart tissue and protected the heart from cyclophosphamide-induced oxidative stress [57]. In this study, we found that squalene could effectively upregulate the expression of *sod* (by 2.5-fold) and *gpx4b* (by 1.3-fold) in the CuSO_4_-induced inflammation model (Figure 4A,B). Compared with the CuSO_4_ treatment alone group, 1% squalene treatment significantly upregulated the expression of *sod* (Figure 4A). Both 0.7% and 1% squalene significantly upregulated the expression of *gpx4b* (Figure 4B). In summary, squalene can significantly upregulate the expression of antioxidant genes *sod* and *gpx4b*, and can effectively reduce the ROS produced in zebrafish. This reduced CuSO_4_-induced oxidative stress, indicating that squalene had an antioxidant effect on zebrafish.

### 3.2. The Anti-Inflammatory Effect of Squalene in the Zebrafish Model

Exposure to copper causes hair cell necrosis in zebrafish lateral line. In addition, it can promote neutrophil migration and accumulation in the inflammatory area, followed by an acute inflammatory response [43,58]. Treatment with *Bacillus coagulans* XY2 before CuSO_4_ exposure significantly reduced neutrophil mobilization, thereby alleviating the acute inflammation induced by CuSO_4_ [59]. Therefore, this study used transgenic *Tg* (*lyz*: *DsRED2*) zebrafish to establish an inflammatory model to study the effect of squalene on neutrophil migration. The results showed that squalene treatment could reduce the number of migratory neutrophils (Figure 5A,B). Among the squalene concentrations, the 0.7% and 1% squalene treatment groups showed the best effects, with no significant difference between them (Figure 5B). In the zebrafish inflammation model, squalene could effectively inhibit neutrophil migration and the inflammatory response, demonstrating a good anti-inflammatory effect. It reduced the number of neutrophils in the inflammatory foci and alleviated the inflammatory symptoms to a certain extent. 

Squalene has been shown to inhibit intracellular oxidative stress in LPS-stimulated mouse macrophages by inhibiting TNFα, inducible nitric oxide synthase (iNOS), COX-2, and NFE2 like BZIP transcription factor 2 (Nrf2) signaling pathways [44,60]. Similarly, squalene downregulated COX-2 and iNOS expression in a mouse acute colitis model [24,28]. There is abundant evidence that certain pro-inflammatory cytokines, such as IL-1β and TNFα, are involved in the process of pathological inflammation. In addition, COX-2 could be induced during tissue damage or inflammation in response to cytokines [61]. Pinostrobin, as a potential anti-inflammatory drug, could down-regulate *Tnfa* and *Cox-2* expression in LPS-stimulated RAW 264.7 macrophages and zebrafish larvae by inhibiting gene expression [62]. Squalene dose-dependently reduced the increased levels of TNF-α and COX-2 in LPS-induced RAW cells [44]. Moreover, addition of squalene to human monocytes and neutrophils activated by incubation with lipopolysaccharide was found to suppress *COX-2*, *IL1β*, and *TNF* mRNA expression [60]. Therefore, in this study, the expression of *tnfa* and *cox-2* was tested in 3 dpf zebrafish treated with 10 μM CuSO_4_ for 24 h to verify the anti-inflammatory effect of squalene. Compared with the control, the expression of *tnfa* and *cox-2* in zebrafish was significantly increased after stimulation with CuSO_4_ (Figure 6A,B). This indicated the regulation of cells after an inflammatory response. However, the expression of *tnfa* and *cox-2* was significantly downregulated after treatment with squalene in a dose-dependent manner. In addition, squalene had beneficial regulatory effect on the post-inflammatory response. In summary, squalene could inhibit the migration of neutrophils to the inflammatory site and inhibit the inflammatory response by downregulating the expression of *tnfa* and *cox-2*, which had an anti-inflammatory effect. These results provide the evidence that squalene exerts its anti-inflammatory activities via mechanisms targeting pro-inflammatory mediators (*tnfα*, *cox-2*) mediators and pathways in closely related phagocytic cells that cooperate during the onset, progression, and resolution of inflammation.

## 4. Materials and Methods

### 4.1. Fish Husbandry

Transgenic *Tg* (*lyz*: *DsRED2*) and wild-type (AB strain) zebrafish (*Danio rerio*) were purchased from the Institute of Hydrobiology of the Chinese Academy of Sciences, China Zebrafish Resource Center (CZRC, Wuhan, China). Zebrafish were maintained and handled according to the Zebrafish Book (zfin.org, 1 June 2022). The zebrafish were fed twice a day. The night before spawning, male and female fish in a 1:1 ratio were transferred to a darkened mating cage. Mating and spawning occurred within 1 h after turning on the lights in the morning. Selected healthy embryos were washed and examined under a microscope. Embryos were housed at 28 °C in embryo-rearing media (E3, see Supplementary Material) (Nanjing EzeRinka Biotechnology Co., Ltd., Nanjing, China). At 3 days post-fertilization (dpf), the larvae were used for the experiments. The components of E3 were NaCl (29.4 g/100 mL), KCl (1.27 g/100 mL), CaCl_2_·2H_2_O (4.85 g/100 mL), and MgSO_4_·7H_2_O (8.13 g/100 mL) [63,64].

### 4.2. Preparation of Test Samples

Squalene (Shanghai yuanye Bio-Technology Co., Ltd., Shanghai, China) exists as an oily liquid at room temperature [17]; therefore, the concentration of squalene is expressed by the volume fraction. Dimethyl sulfoxide (DMSO) (Sangon Biotech Co., Ltd., Shanghai, China) was used as the solvent. The squalene samples were dissolved in 6% DMSO and 90% E3 to form a 4% stock solution. The stock solution was diluted to different concentrations with E3 before use, such that the concentrations of the test sample were 0, 0.05%, 0.2%, 0.5%, 0.7%, and 1%. H_2_DCFDA (Merck Co., Ltd., Shanghai, China) was prepared as a stock of 20 µg/mL in DMSO and frozen at −20 °C. Its working solution was prepared on the day of the experiment.

### 4.3. The Metal Chelating Ability

The CCA was determined using PV (Merck Co., Ltd.) [65]. A total of 30 μL of different concentrations of squalene solution (0.05%, 0.2%, 0.5%, 0.7%, and 1%) or water (control) were mixed with 200 μL 50 mmol/L sodium acetate buffer. Each group had 3 repetitions. Thereafter, 30 μL of 100 mg/L CuSO_4_·5H_2_O solution was added to each group and oscillated for 2 min. After 2 min, 8.5 μL of 2 mmol/L catechol violet solution was added to start the reaction. All the reaction mixtures were shaken at 25 °C for 10 min, and then the absorbance was measured at 632 nm using an ultraviolet-visible (UV-Vis) spectrophotometer [37]. The CCA of squalene was calculated as

Cu^2+^ chelating ability of squalene (%) = (Abs control − Abs sample) × 100/Abs control

where Abs control is the absorbance of the control and Abs sample is the absorbance of the sample.

### 4.4. Antioxidant Capacity In Vitro

DPPH (Sangon Biotech Co., Ltd.) antioxidant capacity was determined according to a previously published protocol [34,37]. A total of 20 μL of squalene solution at different concentrations (0.05%, 0.2%, 0.5%, 0.7%, and 1%) was added to 180 μL of a methanol solution of 0.1 mM DPPH (the ratio was 1:9). Each group had 3 repetitions. Methanol, E3, and DMSO were used as controls, with quercetin as the positive control. After mixing, the reactions were placed in dark at room temperature for 15 min. Then, the absorbance was measured at 517 nm using an UV-Vis spectrophotometer. The calculation formula was

Scavenging Activity (%) = (Abs control − Abs sample) × 100/(Abs control)

### 4.5. Antioxidant Capacity In Vivo

Before the formal experiment, no death or deformity was found in zebrafish soaked with CuSO_4_ or squalene in the experimental concentration range. Quantification of ROS in zebrafish larvae was determined using the cell-permeable fluorescent probe H_2_DCFDA [37]. Zebrafish larvae of at 3 dpf were transferred to a 6-well plate, 10 larvae per well. The zebrafish larvae were treated with 2 mL squalene (0, 0.05%, 0.2%, 0.5%, 0.7%, or 1%) or 100 μM quercetin (positive control) for 1 h. Each group had 4 repetitions. Then, oxidative stress was induced in the zebrafish using CuSO_4_ at a final concentration of 10 µM. After 20 min of exposure, the larvae were washed three times with fresh E3 medium. The larvae were then incubated with H_2_DCFDA solution (20 µg/mL) for 1 h in the dark at 28 °C. The larvae were then washed three times with fresh E3 medium. Zebrafish larvae were immobilized in 3% methylcellulose (Merck Co., Ltd.). The larvae were observed under a fluorescence microscope (Olympus, Tokyo, Japan) and photographed. FI quantification was performed in individual confocal slices using ImageJ software (version 1.6, NIH, Bethesda, MD, USA). Larvae treated with CuSO_4_ alone were used as the negative control, and larvae treated with quercetin were used as positive controls.

### 4.6. In Vivo Neutrophil Recruitment Assay

The *Tg* (*lyz*: *DsRED2*) zebrafish transgenic line was used for the neutrophil recruitment assay in vivo [66]. The 3 dpf zebrafish larvae were treated with 2 mL squalene (0, 0.05%, 0.2%, 0.5%, 0.7%, or 1%) for 1 h (10 larvae per well, and each group had 4 repetitions.). Next, the larvae were treated with 20 µM CuSO_4_ for 1 h. Neutrophil migration was monitored using images taken using a fluorescence microscope (Olympus). Neutrophil migration was quantified by calculating the number of labeled cells detected in the lateral line region. Larvae treated with DMSO alone were used as a control, and larvae treated with CuSO_4_ alone were used as a negative control. 

### 4.7. Real-Time Quantitative Polymerase Chain Reaction (RT-qPCR) Analysis

The expression of selected genes (*sod* (encoding superoxide dismutase), *gpx4b* (encoding glutathione peroxidase 4b), *tnfa* (encoding tumor necrosis factor alpha), and *cox2* (encoding cyclooxygenase 2) was analyzed using RT-qPCR (the primers are shown in Table 1, see Supplementary Material) [37]. The 3 dpf zebrafish larvae were transplanted into a 6-well plate, at 30 larvae per well. The 30 hatched larvae in each group were treated with different doses of 2 mL squalene (0, 0.05%, 0.2%, 0.5%, 0.7%, or 1%) for 1 h. Each group had 3 repetitions. The larvae were then treated with 10 μM CuSO_4_ (a concentration that induced oxidative stress and inflammation in zebrafish without resulting in fish death up to 24 h [14]) for 24 h. The larvae were collected after 24 h and stored at −80 °C for RNA extraction. The total RNA extraction was carried out using the Trizol reagent (Invitrogen, Carlsbad, CA, USA) [67]. RNA integrity was determined by electrophoresis through 1% agarose gels [68]. The RNA was reverse transcribed into cDNA using a cDNA reverse transcription kit (Trans Gen Biotech, Shanghai, China). The cDNA was then used as the template for a real-time quantitative polymerase chain reaction using the following cycling conditions: 95 °C for 10 min, followed by 40 cycles of 95 °C for 30 s, and 60 °C for 30 s. The results were calculated using the 2^−ΔΔct^ method [69] with the *actb* gene (encoding beta-actin) as the control.

### 4.8. Statistical Analyses

Normality of the data and homogeneity of the variances were confirmed using Shapiro–Wilk and Levene tests. The data were subjected to two-way analysis of variance (ANOVA) with squalene and copper exposure as the factors. A probability level of 5% (*p* < 0.05) was considered as significant. Kruskal–Wallis non-parametric one-way ANOVA was used when the normality test failed and differences between groups were determined by the Mann–Whitney test. When non-parametric statistics were used, the data are presented using Tukey’s boxplot (the bottom and top of the box are the minimum and maximum numbers, respectively, for each boxplot).

## 5. Conclusions

This study found that squalene could reduce the production of ROS in a CuSO_4_-induced zebrafish inflammation model, and upregulated the expression of *sod* and *gpx4b* to inhibit oxidative stress. Squalene could also inhibit the migration of neutrophils to inflammatory sites and downregulated the expression of pro-inflammatory cytokine genes *tnfa* and *cox2* to inhibit inflammation. The anti-inflammatory and antioxidant effects of squalene on fish were studied from the aspects of aquatic animal models and gene expression changes. The results provide scientific support for squalene as a feed additive to improve the anti-inflammatory and antioxidant capacity of aquatic products.

## Figures and Tables

**Table 1:** Primers sequences [37].

Gene Name	Forward and Reverse Primer Sequences (5′-3′)	Product Size (bp)	GenBank Accession No.
*actb*	F: CCCCATTGAGCACGGTATTG R: ATACATGGCAGGGGTGTTGA	193	AF057040
*tnfa*	F: CACAAAGGCTGCCATTCACT R: GATTGATGGTGTGGCTCAGGT	227	AB183467
*cox-2*	F: ACAGATGCGCTACCAGTCTT R: CCCATGAGGCCTTTGAGAGA	240	NM_153657.1
*sod*	F: ATGGTGAACAAGGCCGTTTG R: AAAGCATGGACGTGGAAACC	152	NM_131294.1
*gpx4b*	F: TGAGAAGGGTTTACGCATCCTG R: TGTTGTTCCCCAGTGTTCCT	209	BC095133.1

## Data Availability

The raw data supporting the conclusions of this article will be made available by the authors, without undue reservation.

## Supplementary Materials

The supporting information can be downloaded at: https://www.mdpi.com/article/10.3390/ijms24108518/s1.
