# Supplementary material for: Anti-Inflammatory and Antioxidant Properties of Squalene in Copper Sulfate-Induced Inflammation in Zebrafish (Danio rerio)
Source: Int J Mol Sci. 2023 May 10;24(10):8518. doi: 10.3390/ijms24108518 (PMC10218039; doi:10.3390/ijms24108518)

Primer concentrations

The concentration of primer stock solution was 10 M. The concentration in the reaction system was 0.4 M.

Locations of primers

actb

ATAAAGCTGTGACCCACCTCACGCTCAGCATTGTGAGTTTTCAGTGCACGCTGAGAAG
ATCTTCACTCCCCTTGTTCACAATAACCTACTAATACACAGCCATGGATGAGGAAATCG
CTGCCCTGGTCGTTGACAACGGCTCCGGTATGTGCAAAGCCGGTTTTGCTGGAGATGA
TGCCCCTCGTGCTGTTTTCCCCTCCATTGTTGGACGACCCAGACATCAGGGAGTGATGG
TTGGCATGGGACAGAAAGACTCCTATGTGGGAGATGAGGCCCAGAGCAAGAGAGGTA
TCCTGACCCTCAAATACCCCATTGAGCACGGTATTGTGACTAACTGGGATGACATGGAG
AAGATCTGGCATCACACCTTCTACAATGAGCTCCGTGTTGCCCCTGAGGAGCACCCTG
TGCTGCTCACTGAGGCTCCCCTGAATCCCAAAGCCAACAGAGAGAAGATGACACAGA
TCATGTTCGAGACCTTCAACACCCCTGCCATGTATGTGGCCATCCAGGCTGTGCTCTCT
CTGTACGCTTCTGGTCGTACTACTGGTATTGTGATGGACTCTGGTGATGGTGTGACCCA
CACCGTGCCCATCTATGAGGGTTACGCTCTTCCCCATGCCATCCTGCGTCTGGATCTGG
CTGGTCGTGACCTGACAGACTACCTGATGAAGATCCTGACCGAGCGTGGCTACAGCTT
CACCACCACGGCCGAAAGAGAAATTGTCCGTGACATCAAGGAGAAGCTGTGCTACGT
GGCCCTGGACTTTGAGCAGGAGATGGGAACCGCTGCCTCCTCTTCCTCCCTGGAGAAG
AGCTATGAGCTGCCTGACGGTCAGGTCATCACCATTGGCAATGAGCGTTTCCGTTGCCC
CGAGGCTCTCTTCCAGCCTTCCTTCCTGGGTATGGAATCTTGCGGTATCCATGAGACCA
CCTTCAACTCCATCATGAAGTGCGACGTGGACATCCGTAAGGACCTGTATGCCAACAC
AGTGCTGTCTGGAGGTACCACCATGTACCCTGGCATTGCTGACCGTATGCAGAAGGAG
ATCACCTCTCTTGCTCCTTCCACCATGAAGATCAAGATCATTGCTCCCCCTGAGCGTAA
ATACTCCGTCTGGATCGGTGGCTCCATCTTGGCCTCCCTGTCCACCTTCCAGCAGATGT
GGATCAGCAAGCAGGAGTACGATGAGTCTGGCCCATCCATCGTCCACAGGAAGTGCTT
CTAAACAGAACTGTTGCCACCTTAAAATGGCCATGCAATGAGATTCAAACGAACGACC
AACCTAAACCTCTCGAACAAGATGACATCAGCATGGCTTCTGCTCTGTATGGCGCATTG
ACTCAGGATGCGGAAACTGGAAAGGGAGGTAGTTGTCTAACAGGGGGAGGAGCTTTC
CCCGAGAGGACTTCAATGTACATTTCTTTTAGTCATTCCAGAAGCGTTTACCACTTTGC
CCTCCTCACAATGGGCGTCCATGACCTTTTTGTTATAGTGTTTATGTAAATTATGTACTCG
ATACATTGTTTTTCTTTTTGTACTTCAGCCTTAAACTTGGCCCAGTTTGTTATTGTTGCA
ATGAGGGGAAAGCTTTACCTTTTAAAAAGTGAAGATCTTGCAGGACTTCCCTAGGGTA
TGTGAATAAGGGATGTCCCTTGAAAATGTAAGCCAGGGTGTCTCTGTACACTGACAAG
TCAACCCAAATAAAACGTGCACATGTAAAACCACA

tnfα

AAACTCAGTAAAAAAAGGAACACAGCAAGGAGAACTAGCAGCATGGTGAGATACGAA

ACAACATTAGTAGATATGGAAGCCGGTGTCGGGGGAGTTTATCAGACAACCGTGGCAC
CTGTACCGGTTAATTCTTCAAGAAGCTGGATATGGAAGACACTCGCTGCAGTTGCTTTT
GTTGGCCTCTGCGTTGTGGCTGCATTTTTCTTCACCTGGCATGTGATGAAGCCAAACGA
AGAAGGTCAGAAACCCAACAGAGAACATCTAAAACAACAAATCACCACACCTTCAGC
TTCCTCAGACCACGGAAAAGTGCTGAAGCAGATCGCTCAGAGCACAAAGGCTGCCAT
TCACTTGCATGGTGACCCTTCAGGACAATCGCTGAAATGGGTTGGCGGTGTGGATCAG
GCATTCCAACAGGGCGGCCTCAGGCTGGAAAACAACGAGATCATCATTCCTAAGGATG
GTCTTTACTTTGTGTACAGTCAAGTGTCGTACGAAACTTTGTGCGTTGAAGATGTTGAA
GGAGATGGTCAAAAGTACCTGAGCCACACCATCAATCGATACACAGATGCGGTGAGGG
AAAAGATGCCTCTGCAAAACTCGGCCAATTCCGTCTGCCAGTCTCTAGATGGAAAAAC
GTCCTACAGCACCATTTACCTTGGAGCCGTCTTTGATCTTTTTGGGGATGACAGGCTGT
CGACCCACACCACTCGAGTGGGCGACATCGAGAACAACTACGCCAAAACATTCTTCG
GGGTGTTCGCGTTGTGATTTAATAGTCGTGTGGCTCTAGTATGAAAAAACAGTGTTTTA
TGTTAAAACATTGAATGTATGTTCTCAGGGCAAGAAATTCGACTAAGGCTAAGAGGCC
TCCCTGCATCGTGATGACTTGTTTTATATGTAAAACAAAGCCGATGCAGTGAGAGTTGT
GCACTGAATACCATGTAGTCAGTCAAGAGCACTATAACAAGGGCACTACAATTTGTGTA
CTTATCGCAAGCTAATGTGACGTTTGATTATCTTACGTATTTCGTTGTAGAAATAAGTAG
ACTTTTATTTTATTTTTGCTGTTGTATTGATCTATTTATAACTTTTACACCTATTTATATATT
GTATTTGTTGTATTTATCTATTTATTAATGACTCCATGCTGTTATTTGCCGAATGAATGTAA
TGAAATGTGTTTCCGACACTCCTCTGTGATTTTAAAAATAAATCAA

cox2

AATACAACGGGCTCGACCAGAGTAACGCATCTCTGCTTCAACAGCCTCTAGATAATAAC
ACTTCTGGAATGAATAAACTGGTTTGCTTGGTCCTACTTTCAAGCATTTGGATCTTTCCT
GGTGAAGGATACGACCCTTGCTGTGCACAGCCTTGCCAAAACCAGGGCGTGTGTTTAT
CCAAGGGAGCTGATGCTTATGAATGTGACTGCACCAGGACAGGATATTATGGTGAAAA
CTGCACTACCCCTGAGCTTCTCACACGCATCAAATCAGCTCTGAAACCAAGACCAAAT
GTAGTGCATCATATTTTAACACATTATAAATCAATCTGGGATATCATCAACAGCATCTCTT
ACCTGAGGGATGGCATAATGCGCTATATCCTGTTGTCAAGGTCCCATTTGGTTGAAAGT
CCACCGACGTACAATGCTGATTATGGCTACAAAAGCTGGGAAGCGTACTCTAATTTGTC
CTATTACACCCGCACCCTTGCCCCGTTGCCCCAAAACTGCCCAACGCCTGATCTCCCAA
ATGCCAAGCAGGTGGTGGAGCAGGTGCTGTTGAGGAAGCAGTTCATCCCTGATCCACA
GAGGAGCAGTCTCATGTTTGCTTTCTTCGCCCAGCATTTCTCTCACCAGTTCTTCAAGT
CGGACTTTAAAAAGGGACCAGCCTTCACCAAAGCCCTGGGTCATGGAGTGGATCTGG
GGCACATTTATGGAGAGACGCTGGAGGTTCAACACAAACTTCGTCTGTTTAAAGATGG
AAAGCTTAAATACCAGGTTGTGGATGGTGAGGTGTACCCTCCGCTTGTGAAGGACGTC
CAGGTGGAGATGCATTACCCTCCTCATATCCCGGAAGAGCAGAAATTTGCTGTGGGCC
ATGAGGCCTTTGGTCTGGTTCCAGGTTTGATGATGTATGCTACCATTTGGCTCCGTGAG
CACAACCGTGTCTGTGACATCATGAAGCAGGAACATCCTGACTGGGATGATGAGAGAA
TCTTCCAAACCACTCGTCTCATCCTGATTGGTGAGACCATCAAAATTGTGATCGAGGAC
TATGTTCAGCACTTGAGTGGATACAACTTCAAGCTCAAGTTTGACCCAGAGCTCATCTT
CAGTGAGCGTTTCCAGTACCAGAACCGTATTGCGGCCGAATTCAATACCCTGTATCACT

GGCATCCACTGTTGCCGGACAACTTTCAGATCCAGGATCAGATCTACGGCTATCACCAG
TTTGTGTTTAACAACTCTATCGTCACCACACATGGCATCCGCAACATGGTGGACTCCTT
CACCAAACAGACTGCTGGACGGGTGTCTGGTGGACGTAACCTGCCACCTGCAGTTCA
AGGAGTCGCTGTTAAAGTTCTGGAACAAACCAGACAGATGCGCTACCAGTCTTTCAAT
GCATACAGAAGACGCTTCAACATGAAGCCCTACTCATCCTTTGAGGAGATGACAGGAG
ACAAAGATCTGGCTGCCCAGCTGAAGGAGCTGTATGGTCACGTAGACAAGGTTGAGCT
GTATCCTGGCCTTCTTGTGGAGAAATCCAGACCCAACTCTGTGTTTGGGGAGACTATGG
TGGAAATGGGAGCTCCCTACTCTCTCAAAGGCCTCATGGGAAATGCTATTTGTTCTCCT
GAATACTGGAAGCCCAGCACATTTGGTGGGAAAGTGGGATTTGACATCGTTAACTCTG
CTTCACTGAAGAAATTGGTGTGTTTGAACATCAGTGGGCCCTGTCCGATGGTGTCCTTT
CAGGTGCCAGATGTGAAATTTCAGAGTTCAGAAAACGTGAATTCAAGTTCAGTGCACT
CCACAGTAAATAATATAAATCCAACTGTTGTTTTGAACGAGCGGAGTTCAGAGCTGTAA
AAGGTCTCAAAATCTTCAAAATCCCAGATACTTGATATGGTTTTGTAATTTATTTATTTTT
TATATATATTTATTTGAATAATTTATTGCAGTAGTGTATTTATTGATTGTTTTTTTTTTTGTA
TTGTGATCGATGTGCAACTTTGTTTCATAAATTCATGTGAGGTATTCGACTAATTGGACA
AACACATTTCAAATGCCAATCAACTGTTGCAAATGTTTATTTCATAAATAAAAACAAAT
GATATTTTGCAAAAATAAAAAAAAAAAAAAAAA

sod

TCTTATCAAACACAGTCGGTTTCTTTCACTCTCTCACAACTTCTCAGTTTGCATAATCTA
CAGTCAGCATGGTGAACAAGGCCGTTTGTGTGCTTAAAGGCACCGGTGAAGTGACCG
GCACCGTCTATTTCAATCAAGAGGGTGAAAAGAAGCCAGTGAAGGTGACTGGTGAAA
TTACTGGCCTTACTCCAGGAAAACATGGTTTCCACGTCCATGCTTTTGGTGACAACACA
AACGGCTGCATCAGTGCAGGTCCGCACTTCAACCCTCATGACAAAACTCATGGTGGGC
CAACCGATAGTGTGAGACACGTCGGAGACCTGGGTAATGTGACCGCTGATGCCAGTGG
TGTTGCAAAAATTGAAATCGAGGATGCAATGCTAACTTTGTCAGGCCAACATTCTATTA
TTGGGAGGACCATGGTGATTCATGAGAAGGAGGATGACTTGGGGAAGGGTGGCAATG
AGGAAAGTCTTAAAACTGGCAACGCTGGCGGTCGTCTGGCCTGTGGAGTGATCGGCAT
CACTCAGTGAATCTGCTCTAATGGAAGAGCCGGTTGAAATATTGGTGACCAATGTGGAT
GCCTCTGAAAGCACTTAGCCCGCTGACAATTACATCTCTATTTTTGTTAGGTAACGTTG
AAAGAATTGTACTTTAGCCTTGTTAAGCTGTTTTTCTGTGCATTTCTATGTGGGCAACC
TTTTATTTGTTATCTGCTTAATTCATTAAACATTGAGCAGACTGGAAAAAAAAAAAAAA
AA

gpx4b

GCTACTAGTATGTGGTTGTTTCAGAGAGCGCTGTTGGTCGGAGCGGTGGGCAGCAAGA
GCTTCGCCAGAGCAATGTGTGCCCAAGCCAATGACTGGCAGTCGGCCAAATCCATATA
CGAGTTCTCAGCCATAGACATTGATGGAAATGATGTTTCTCTGGAGAAATACAGGGGTT
ATGTGTGTATCATCACAAACGTAGCCTCTAAATGAGGTAAAACTCCAGTAAACTACACT
CAGCTTGCGGCAATGCACGTCACGTACGCTGAGAAGGGTTTACGCATCCTGGGCTTCC

CCTGCAACCAGTTCGGAAAGCAGGAGCCTGGAAGTGAAGCGGAGATTAAGGAGTTTG
CTAAAGGCTACAATGCAGAGTTTGATCTGTTCAGTAAGATCGATGTGAACGGAGACGC
AGCTCATCCTCTCTGGAAGTGGATGAAGGAGCAGCCCAAAGGCAGAGGAACACTGGG
GAACAACATCAAGTGGAATTTCACCAAGTTCCTCATTGATAGAGAGGGTCAGGTTGTG
AAGAGGTACGGACCAATGGACGATCCAAGCGTGGTGGAGAAGGATCTTCCCAAGTAC
CTGTAGAGTGTGTATGCTGCGGCGTCTCCGTCCTGTGTTTTGGGCTCTGTTCTCTCTGG
AGCAGAAACGCTGGACGTCTGCAGGTGAAGCCCAGACGTGTGACGGCTGATCCTCAA
GCCTGTGTTCAGGTGGGTTCGGCCTGATGTTCCCGACGTGCAAAAATACTGGGAAAAC
CCTGCAAATGTTCAGACAAGTGTGTCCAGAGAGGAGCTTCATGCACTGAAAATACACT
GAGCAATCTTTTATAATCAACAGCCATAATTAAAGAGTCATGTCATCAAAAAAAAAAAA
AAAAAA

Components of E3

The components of E3 were: NaCl (29.4 g/100 mL), KCl (1.27 g/100 mL), $CaCl_2 \cdot 2H_2O$ (4.85 g/100 mL), and $MgSO_4 \cdot 7H_2O$ (8.13 g/100 mL).

The structural formula of squalene

Chemical formula: $C_{30}H_{50}$

Molecular weights: 410.718.

Structural formula:

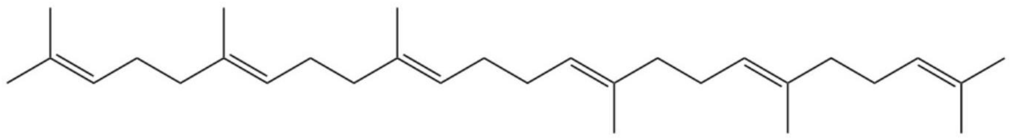

Supplement: Supplementary file 1 [file ijms-24-08518-s001.zip › ijms-2292203-supplementary.pdf]
